# Temporary severe bradycardia due to pacemaker programming

**DOI:** 10.1007/s12471-016-0855-1

**Published:** 2016-06-09

**Authors:** A. Böhm, R. G. Kiss, P. Bogyi, G. Z. Duray

**Affiliations:** Department of Cardiology, Military Hospital, Budapest, Hungary

A 67-year-old male patient received a pacemaker for syncope. His parameters at check-up were: P/R amplitude 2.8/11 mV and atrial/ventricular threshold 0.875/0.625. The pacemaker was programmed as follows: lower rate 60 bpm; upper tracking rate 130 bpm; sensed AV interval 120 ms; paced AV interval 50 ms; atrial/ventricular sensitivity 0.5/5.6 mV, and atrial/ventricular output 2/2 V. Due to syncope in his history, the rate drop response was also activated: drop size 25 bpm; drop rate 50 bpm; detection beats: 2 beats. Both with an intervention rate of 100 bpm.

The following ECG was recorded during carotid massage (Fig. 1). What is the explanation for this interesting ECG recording?

**Fig. 1:**
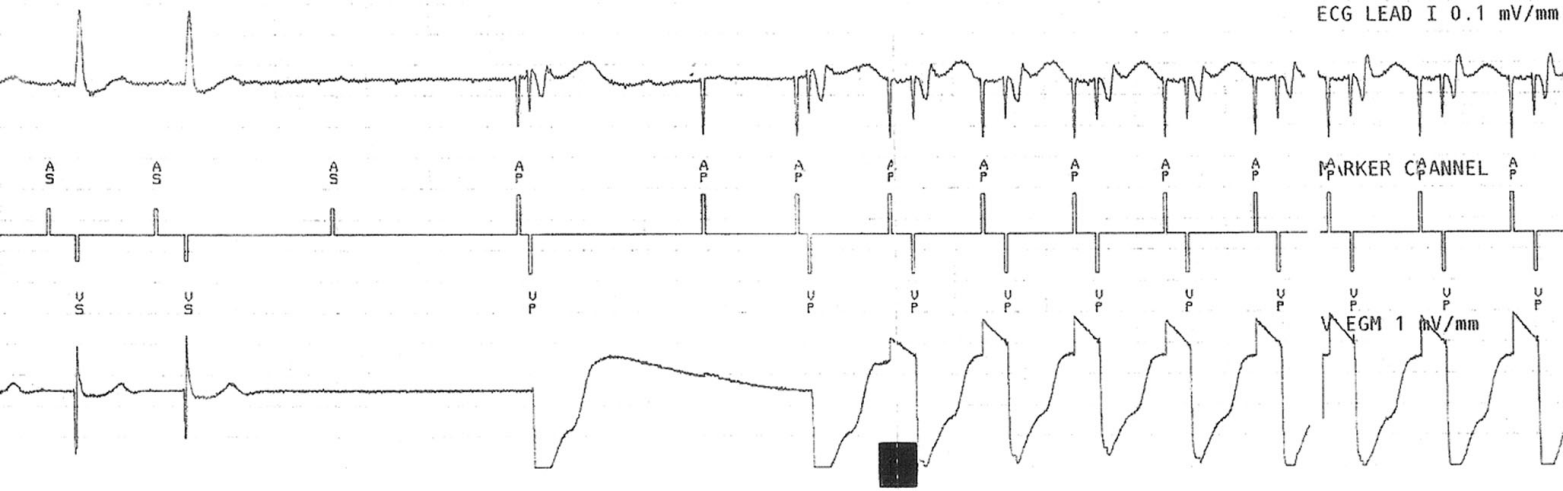
ECG recording during carotid massage: Lead I, marker channel, ventricular EGM

## Answer

You will find the answer elsewhere in this issue

